# Diffusion Tensor Imaging Before and 3 Months After Concentrated Exposure Response Prevention in Obsessive-Compulsive Disorder

**DOI:** 10.3389/fpsyt.2021.674020

**Published:** 2021-05-26

**Authors:** Vilde Brecke, Anders Lillevik Thorsen, Olga Therese Ousdal, Chris Vriend, Dag Alnæs, Kristen Hagen, Bjarne Hansen, Gerd Kvale, Odile A. van den Heuvel

**Affiliations:** ^1^Bergen Center for Brain Plasticity, Haukeland University Hospital, Bergen, Norway; ^2^Centre for Crisis Psychology, University of Bergen, Bergen, Norway; ^3^Department of Clinical Psychology, University of Bergen, Bergen, Norway; ^4^Department of Radiology, Haukeland University Hospital, Bergen, Norway; ^5^Department of Psychiatry, Department of Anatomy and Neurosciences, Amsterdam Neuroscience, Amsterdam UMC, Vrije Universiteit Amsterdam, Amsterdam, Netherlands; ^6^Bjørknes College, Oslo, Norway; ^7^Norwegian Centre for Mental Disorders Research (NORMENT), Division of Mental Health and Addiction, Oslo University Hospital and Institute of Clinical Medicine, University of Oslo, Oslo, Norway; ^8^Psychiatric Department, Hospital of Molde, Molde, Norway

**Keywords:** obsessive-compulsive disorder, exposure and response prevention, white matter microstructural integrity, diffusion tensor imaging, tract-based spatial statistic

## Abstract

**Background:** Subtle differences in white matter microstructure have been found in obsessive-compulsive disorder (OCD) compared to controls using diffusion tensor imaging (DTI), but it is unclear if and how this change after treatment. The primary aim of this pre-registered study was to investigate white matter integrity between OCD patients and controls and changes after concentrated exposure and response prevention (ERP).

**Methods:** Fractional anisotropy (FA), radial diffusivity (RD), axial diffusivity (AD) and mean diffusivity (MD) were estimated using FMRIB Software Library (FSL). The images were registered to a study-specific template using a longitudinal pipeline based on full tensor information in DTI-TK. Voxel-based analysis was performed using tract-based spatial statistics (TBSS). Using SPSS, we compared the integrity in three bilateral regions of interest (ROI), the sagittal stratum, posterior thalamic radiation and cingulum, in 32 OCD patients and 30 matched healthy controls at baseline. Patients received a four-day concentrated ERP format. We investigated longitudinal changes in 26 OCD patients and 22 healthy controls at 3months follow-up using repeated-measures ANOVA. Exploratory *t*-tests were conducted for AD and MD. Secondary hypothesis used linear regression to investigate if baseline FA predict treatment outcome 3 months later, and if patients with illness onset before 18 years of age would show lower FA in sagittal stratum. Finally, we performed sensitivity analysis on medication and comorbidity influences on FA.

**Results:** Three months after treatment, 77% of the patients were in remission. Contrary to our hypotheses, we did not find any significant differences in FA, RD, AD or MD between the groups before treatment, nor significant group by time effects in any of the ROI. None of the baseline FA measures significantly predicted treatment outcome. Illness onset before 18 years of age did not significantly predict FA in the sagittal stratum. Adjusting for medication or comorbid anxiety or mood disorder did not influence the results.

**Conclusions:** Although concentrated ERP in OCD lead to high remission, we did not find significant long-term changes by DTI. Future studies will benefit from using larger sample sizes and multi-shell diffusion-weighted imaging when investigating white matter microstructure in OCD and underlying neurobiological mechanisms of treatment.

## Introduction

Obsessive-compulsive disorder (OCD) characterized by intrusive, recurrent mental obsessions followed by various compulsive responses performed in the attempt to neutralize the discomfort (1), with a world-wide estimated prevalence of up to 2% combined with high rates of comorbidity (2). OCD is associated with abnormalities in the function and structure of cortico-striato-thalamo-cortical, fronto-limbic and fronto-parietal circuits (1). Diffusion tensor imaging (DTI) allows for modeling of white matter microstructure in white matter tracts connecting different regions and circuits in the brain (3, 4). Emerging results based on DTI data indicate that several white matter tracts may show lower integrity in OCD patients compared to controls as reflected in the measure of fractional anisotropy (FA) [e.g. (5)]. The FA value ranges from 0 to 1 and indicates the average diffusivity restriction in the voxel (6). Common DTI measures include mean diffusivity (MD), which is the average from all three of the tensor eigenvalues. Axial diffusivity (AD) is sensitive to the longest eigenvalue, while radial diffusivity (RD) represents the two shortest eigenvalues (6). Regarding the findings of white matter alterations in OCD, it remains to be determined if white matter microstructure features are stable, potentially underlying trait characteristics that contribute to the risk of developing OCD [e.g., (7)], or if they normalize once the patients recover [e.g., (8, 9)]. Longitudinal studies on white matter microstructure before and after successful treatment are therefore needed to better understand the pathophysiology of the disorder and the potential for treatment-related change.

Studies using DTI in OCD suggest that several white matter tracts may be affected in the disorder. The Enhancing NeuroImaging Genetics through Meta-Analysis (ENIGMA) OCD Working Group used harmonized image processing and tract-based spatial statistics on DTI data from 700 adult patients, 645 adult controls, 174 pediatric patients and 144 pediatric controls from multiple sites (5). Using meta-analysis, the authors reported lower fractional anisotropy (FA) in the posterior thalamic radiation and sagittal stratum in adults with OCD. OCD patients also showed higher RD in these regions, but this did not survive correction for multiple comparisons. No significant group differences were found for AD or MD. Lower FA in the sagittal stratum in adult OCD patients was associated with younger age of illness onset, longer illness duration, and a higher percentage of medicated patients in the included cohorts. These findings could either indicate an illness specific trait or might be caused by long-term living with the illness (5). A whole-brain meta-analysis of studies using voxel-based analysis (VBA) on white matter volume and FA found the most pronounced alterations (increased volume and reduced FA) in parts of the corpus callosum body and cingulum, primarily in adult OCD patient (10). Recent studies have further corroborated the finding of lower FA in the cingulum (11–13) along with findings of higher radial diffusivity (RD) (12, 13).

Cognitive-behavioral therapy (CBT), including exposure and response prevention (ERP), are effective treatments for OCD (14–16). Previous studies combining neuroimaging and CBT in OCD have found changes after treatment in gray matter volume, resting-state connectivity, and brain activation during symptom provocation, as well as in glutamate levels or other spectroscopy derived neurometabolites [e.g., (17–24)]. However, the findings are heterogeneous and often not replicated. Only one previous study has applied DTI before and after CBT. In this study, FA increased in medial and ventral prefrontal regions, medial temporal gyrus, and decreased RD in the right posterior internal capsule after 12 weeks of CBT in 56 unmedicated OCD patients (25). One SSRI treatment study found decreased RD in the left stratum after 12 weeks in 27 patients (8), while another with 13 patients found decreased FA in the posterior thalamic radiation (9). Together, these treatment studies suggest that clinical improvement may be related to changes in white matter microstructure, but the findings are inconsistent regarding the location, magnitude and direction of changes after treatment.

In the present study, we first compared FA values between OCD patients and demographically matched healthy controls the day before treatment. Patients took part in concentrated ERP over four consecutive days, called the Bergen 4-Day Treatment (B4DT), while healthy controls received no intervention. Both groups were re-scanned after 3 months, which provided an opportunity to detect white matter changes after a period of normalized living when most patients were in remission. Based on previous findings (5, 8–10, 25), we hypothesized to find lower FA in the cingulum bundle, sagittal stratum, and posterior thalamic radiation at baseline in OCD patients vs. healthy controls, along with higher RD in the same regions. We expected OCD patients to show an increase in FA in the cingulum, sagittal stratum, and posterior thalamic radiation 3 months after treatment, while we expected no changes in the healthy controls. We also expected that these tracts would show decreased RD after treatment in OCD patients. Changes in FA and RD were expected to be related to improvements in symptom severity. Finally, we hypothesized to find lower FA in the sagittal stratum of patients with illness onset before the age of 18 compared to patients with onset after 18 years (5). The hypotheses and analyzes plan were preregistered at the Open Science Foundation (https://osf.io/vufg8). We performed exploratory analyzes of all regions in the JHU atlas at baseline and after 3 months to explore potential group differences in regions outside the regions of interest, and to allow for future meta-analysis. We also explored if baseline FA and RD in the regions of interest predicted change in Y-BOCS 3 months after treatment using linear regression.

## Materials and Methods

### Sample

Thirty-five patients were recruited from a specialized outpatient clinic at Haukeland University Hospital, Bergen, Norway. Thirty-one diagnosis-free controls were recruited *via* bulletins and email from the local community. The inclusion criteria were 18 years or older, fluency in Norwegian, no known neurological conditions, and for patients, a primary diagnosis of OCD with a score ≥16 on the Yale-Brown Obsessive-Compulsive Scale (Y-BOCS) (26). Patients were excluded if primary symptoms were substance abuse, hoarding, active bipolar or psychosis symptoms, suicidal ideation, intellectual disability, or unwillingness to refrain from psychoactive substances such as benzodiazepine and/or alcohol before or during therapy, as well as contraindications to MRI. All OCD patients were offered the treatment as part of ordinary public mental health care. The study was approved by the Regional Ethics Committee for South-Eastern Norway (2015/936) and all participants provided informed written consent before participation in line with the declaration of Helsinki.

**Figure 1:**
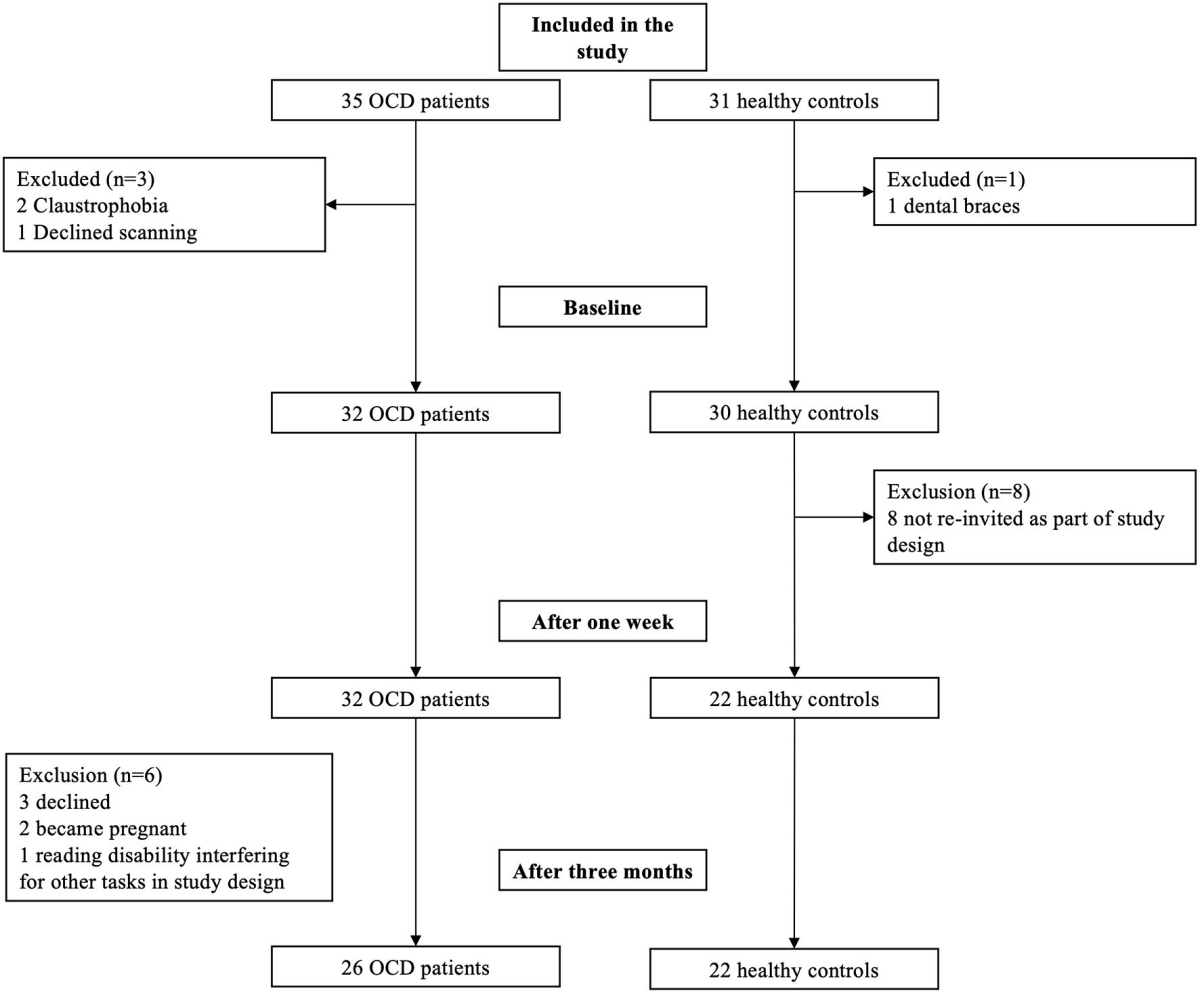
Flowchart of participation over the timespan of the study. This includes from pre-study exclusion to baseline, 1 week after treatment, dropouts, and 3 months after treatment scanning.

The final sample included 32 OCD and 30 healthy controls at baseline, and 26 OCD patients and 22 controls at follow up (see Figure 1). Two patients dropped out from the first scanning because of claustrophobia and one declined the diffusion-weighted imaging. At follow-up, three patients declined further scanning, two were pregnant and one was excluded from further scanning due to reading impairment that interfered with cognitive testing not part of the present study. Eight of the controls were not invited back for longitudinal scanning, in line with planned study design.

### Clinical Assessment

All patients were diagnosed using the Structured Clinical Interview for DSM-IV Axis I Disorders (SCID-I) (27). Self-reports of depressive symptoms were measured with the Patient Health Questionnaire (PHQ9) (28), anxiety symptoms were measured using the Generalized Anxiety Disorder (GAD-7) (29). Obsessive-compulsive symptom severity in patients was assessed using Y-BOCS (26) by trained raters. A total Y-BOCS score below 13 was used as the cut-off for clinical remission after treatment, while a reduction of 35% or more indicate clinical response (30).

### Treatment

The patients underwent a concentrated ERP treatment (ERP) format termed the B4DT program. In this format, the first day of treatment is allocated to psychoeducation and preparation, followed by 2 days of ERP in various contexts, interspersed with group meetings. The last day consist of summarizing the treatment, planning how the patient can integrate the change into their everyday life, and relapse prevention. On the third evening, family and friends are invited for a lecture on how to support the patient in the future. Trained therapists deliver this over four consecutive days with a 1:1 ratio between patients and therapists. The results in clinical practice and randomized controlled trials suggest a remission rate of around 70% (31–34), and recovery rates are retained 4 years after treatment (35).

### MRI Acquisition

We performed scanning on a 3T General Electric Discovery MR750 (GE Healthcare, Milwaukee, Wisconsin, USA) using an eight-channel head coil at Haukeland University Hospital, Bergen. We performed single shell diffusion weighted imaging (DWI) with 30 diffusion-weighted (*b* = 1,000 s/mm^2^) and six non-diffusion-weighted volumes (b = 0 s/mm^2^). Images were acquired using a 128 × 128 matrix, TR = 14s, TE = 90 ms, flip angle = 90°, 51–69 slices depending on head size, slice thickness = 2.4 mm, slice gap = 0 mm, in-plane resolution = 1.72 × 1.72 mm.

#### Diffusion MRI Processing

**Figure 2:**
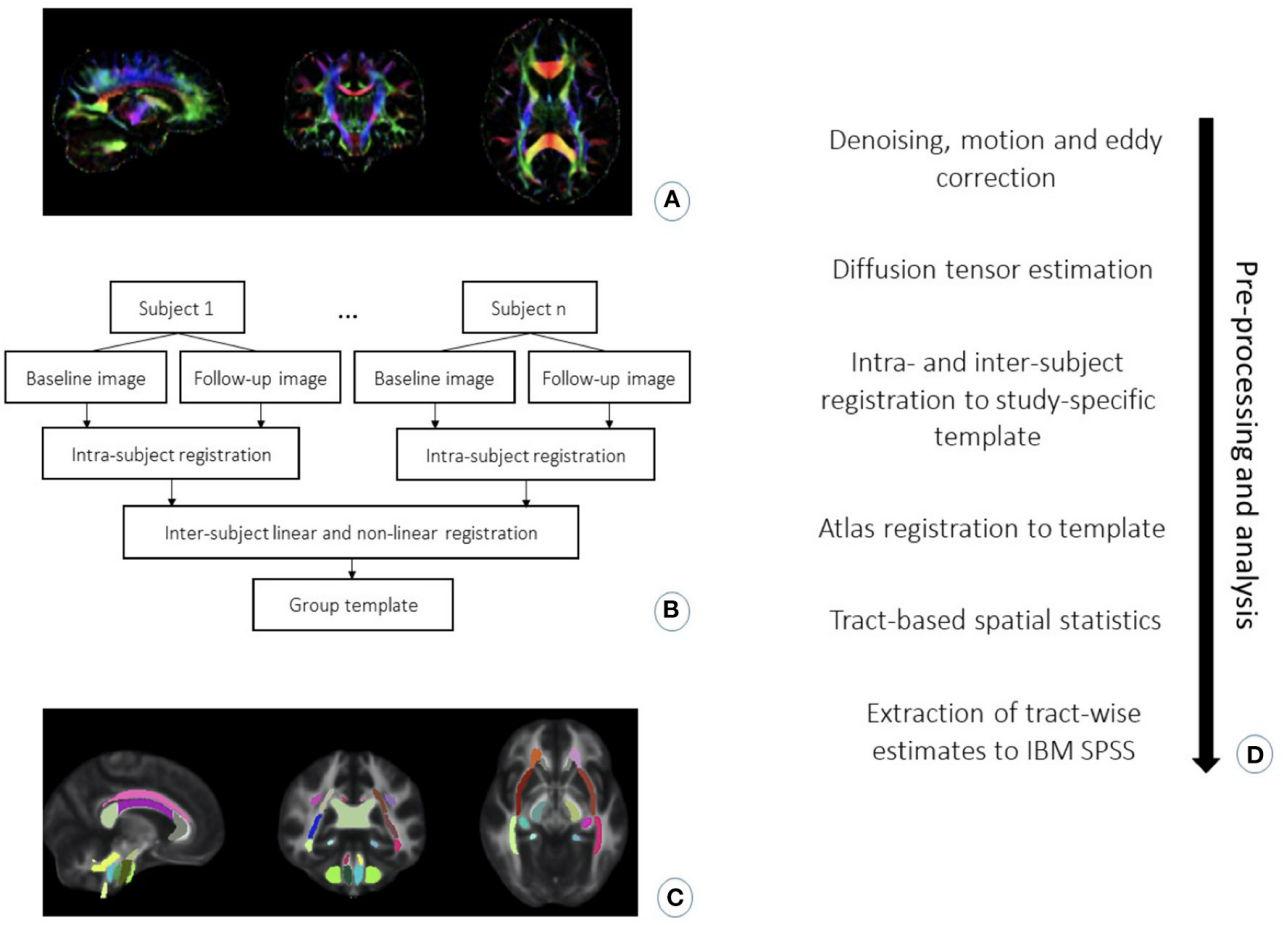
Preprocessing and analysis steps: **(A)** Image of principal diffusion direction from diffusion tensor estimation. **(B)** Flowchart for the pipeline for registration of the DTI data into a study-specific template to align the images onto a common space. **(C)** Visualization of JHU atlas registration onto a study template, which was further used to locate the regions of interest. **(D)** Chronologically ordered listing of pre-processing and analysis steps used in the current study.

The diffusion-weighted data were first denoised using MRtrix (36), followed by corrections for motion within and between volumes and eddy-current induced distortion (37, 38) in FMRIB Software Library [FSL, version 6.0.1; (39)]. The images were visually quality controlled for artifacts and abnormalities. FA, RD, AD and MD maps were computed by fitting a diffusion tensor model to the corrected diffusion data using FSL DTIFIT, followed by visual quality control of the fit of the principal diffusion direction (λ1). DTI-TK was used to non-linearly register DTI images using full tensor information following a protocol developed by Keihaninejad and colleagues (40, 41). This involved generating a study-specific template by first registering each subject's DTI images from each time point to each other and then calculating a mean image of the two. The mean images were used to create the study template. In cases where participants had no follow-up data, we used the baseline scan in the template's creation. Each native image per subject was then diffeomorphically registered to the common study-specific template. Tract-based spatial statistics [TBSS; (42)] was used to create a mean skeleton representing the locally maximal value (43) and threshold was set to FA > 0.2. Each participant's FA, as well as AD, MD and RD data was then projected onto the skeleton. To define the ROIs, FSL FNIRT was used to non-linearly register the Johns Hopkins University (JHU) ICBM-DTI-81 white-matter labels atlas (JHU-ICBM-labels-1 mm) to the study template, as recommended by Mahoney and colleagues (44). The registered atlas was eroded by 2 mm for optimal overlap with the major white matter tracts, which was visually inspected for all participants. We when created a binary mask containing the bilateral dorsal and ventral cingulum bundle, sagittal stratum, and posterior thalamic radiation from the JHU atlas (see Figure 2).

#### Statistical Analysis

Tract-wise differences in FA, RD, MD and AD between the groups were tested using non-parametric permutation tests in randomize (45). Statistical threshold was set at family-wise corrected *p* < 0.05 using threshold-free cluster enhancement (TFCE) with 5,000 permutations (46). We compared the FA, AD, MD, and RD in the ROIs between OCD patients and healthy controls at baseline and 3 months follow-up using two independent *t*-tests with age and sex as covariates. We used linear regression in FSL's randomize to investigate if FA or any of the other diffusion values at baseline were predicted by clinical change. This was modeled using the mean DTI values as the dependent variables and mean centered Y-BOCS score after 3 months, centered pre-treatment-YBOCS score, and centered age added as covariates.

We extracted the mean values for FA, RD, MD, and AD from all regions of the JHU atlas for further analyzes in IBM SPSS Statistics version 26. Here, we examined the differences in regional FA, RD, MD and AD between OCD patients and controls by independent sample *t*-tests at baseline and at 3 months follow-up, and calculated Cohen's *d* as a standardized effect size (see results for FA in Supplementary Material). For each ROI we then used a two (group: OCD vs. HC) by two (time: baseline vs. 3 months follow-up) repeated-measures ANOVA to analyze the main effects and the group by time interactions in 26 OCD patients and 22 healthy controls, along with age and sex as covariates. Partial eta squared ($\eta_{\text{p}}^{2}$) was calculated to show the explained variance by each contrast. We applied the Benjamini-Hochberg False Discovery Rate correction (FDR) per contrast in the repeated-measures ANOVAs to reduce the type I error rate. For paired *t*-tests, we calculated Cohen's d as $\frac{Mean\;1-Mean\;2\;}{SD\;1}$ (47). We used independent samples *t*-tests to compare the mean FA in the bilateral sagittal stratum for patients with a childhood-onset of OCD (<18 years) vs. adult-onset (≥18 years) of disease. For the 26 OCD patients with data at both time points, we used a linear regression to explore if OCD onset was related to changes in FA in the sagittal stratum over time. We used linear regression to investigate the relationship between change in FA and RD in the ROIs and change in Y-BOCS from baseline to 3 months after treatment, co-varying for baseline Y-BOCS scores. The *p*-values for the coefficients for change in FA and RD were separately corrected using FDR. Four patients and two healthy controls had some missing clinical measures (see Supplementary Materials for details). Missing data were estimated using expectation maximization with 25 iterations (See more information on missing values in Supplementary Material). Available information across all three time points were used to replace the missing values (48).

## Result

### Demographics and Clinical Characteristics

OCD patients (*n* = 32) and healthy controls (*n* = 30) were matched on age, sex, handedness, and education (Table 1). The mean age of the OCD patients was 30.25 (SD = 9.01) with 62.5% females. The mean age of the controls was 31.03 (SD = 10.50) and 63.3% females. Before treatment, eight (25%) of the patients were using medication, and all continued using medications throughout participating in the study (see Table 1 for all medications). Fifteen (46.9%) had a comorbid anxiety disorder and eleven (34.4%) had a comorbid mood disorder (see Table 1 for details). Fourteen (43.8%) of the patients were diagnosed with OCD before the age of 18.

**Table 1:** Demographics and clinical characteristics in the sample.

**Characteristic**	**OCD (n=32)**		**HC (*****n*** **=** **30)**		**Statistical Analysis**		
	**Mean**	**SD**	**Mean**	**SD**	***t***	**df**	***P***
Age	30.25	9.01	31.03	10.50	0.32	60	0.75
Years of education	14.53	2.38	14.50	2.33	−0.05	60	0.96
Y-BOCS total score at baseline	27.06	3.93	-	-	-	-	-
Y-BOCS total after treatment	11.41	6.43	-	-	-	-	-
Y-BOCS total at 3M follow-up	10.57	6.39	-	-	-	-	-
PHQ9 Baseline (26 OCD vs. 22 HC)	10.77	5.87	2.62	1.79	-	-	-
PHQ9 One week after (26 OCD vs. 22 HC)	8.37	5.72	2.38	1.93	-	-	-
PHQ9 Follow-up (26 OCD vs. 22 HC)	8.63	6.00	2.15	1.53	-	-	-
GAD7 Baseline (26 OCD vs. 22 HC)	13.06	5.25	2.10	2.55	-	-	-
GAD7 One week after (26 OCD vs. 22 HC)	12.38	5.44	2.10	2.50	-	-	-
GAD7 Follow-up (26 OCD vs. 22 HC)	7.32	4.51	1.95	2.01	-	-	-
	**n (of 32)**	**%**	**n (of 30)**	**%**	**χ**^**2**^	**df**	***P***
Female	20	62.5%	19	63.3%	0.005	1	0.95
Right-handedness	30	93.8%	28	93.3%	0.004	1	0.95
University/College degree	13	40.6%	17	56.7%	2.50	2	0.29
	**n (of 32)**	**% of OCD**					
Medicated at first scanning	8	25%	-	-	-	-	-
SSRI	7	21.9%	-	-	-	-	-
Antipsychotics	1	3.1%	-	-	-	-	-
Ritalin/Methylphenidate	1	3.1%	-	-	-	-	-
Comorbid disorder baseline	15	46.9%	-	-	-	-	-
Comorbid mood disorder	11	34.4%	-	-	-	-	-
Major depressive disorder	10	31.3%	-	-	-	-	-
Dysthymia	2	6.3%	-	-	-	-	-
Social anxiety disorder	7	21.9%	-	-	-	-	-
Comorbid anxiety disorder	15	46.9%	-	-	-	-	-
Generalized anxiety disorder	9	28.1%	-	-	-	-	-
Specific phobia	4	12.5%	-	-	-	-	-
Panic disorder	3	9.4%	-	-	-	-	-
Agoraphobia	3	9.4%	-	-	-	-	-
Hypochondriasis	3	9.4%	-	-	-	-	-
PTSD	1	3.1%	-	-	-	-	-
ADHD	1	3.1%	-	-	-	-	-
Somatization disorder	1	3.1%	-	-	-	-	-
Pain disorder	1	3.1%	-	-	-	-	-
No comorbidity	9	34.6%	-	-	-	-	-
Childhood onset	14	22.6%	-	-	-	-	-

*ADHD, attention deficit hyperactivity disorder; HC, healthy controls; GAD7, generalized anxiety disorder questionnaire; OCD, obsessive-compulsive disorder patients; PHQ9, patient health questionnaire; PTSD, post-traumatic stress disorder; SSRI, selective serotonin reuptake inhibitors; Y-BOCS, yale-brown obsessive-compulsive scale*.

A repeated-measures ANOVA of Y-BOCS scores in the OCD patients showed a significant effect of time (*F*_(1.636,40.894)_ = 129.148, *p* < 0.001, $\eta_{\text{p}}^{2}$ = 0.838) (Table 1). Paired *t*-test in the OCD patients showed a large decrease in symptom severity 1 week after treatment (*t*_(25)_ = 12.893, *p* < 0.001, 95%CI [14.026, 19.359], *d* = 3.92), with no significant change between 1 week and 3 months after treatment (*t*_(25)_ = 0.131, *p* = 0.897, 95%CI [−1.699, 1.930], *d* = 0.16) (Table 2). Three months after treatment, 20 (76.9%) patients were in remission, three (11.5%) responded, and three (11.5%) showed no clinically significant change.

**Table 2:** Repeated measures ANOVA results for mean FA in OCD patients (*n* = 26) and healthy controls (*n* = 22).

	**Time**			**Group**			**Time by group**			**Age**			**Gender**		
**ROI**	***F***	**FDRp**	$\eta_{\text{p}}^{2}$	***F***	**FDRp**	$\eta_{\text{p}}^{2}$	***F***	**FDRp**	$\eta_{\text{p}}^{2}$	**FDRp**	$\eta_{\text{p}}^{2}$	***F***	**FDRp**	$\eta_{\text{p}}^{2}$	
PTR R	0.04	0.79	1.00	0.00	0.99	0.00	0.34	0.90	0.01	11.18	0.99	0.20	0.04	0.97	0.00
PTR L	0.23	0.84	0.01	0.01	0.99	0.00	1.08	0.81	0.02	4.81	0.22	0.10	1.75	0.77	0.04
SS R	0.00	0.85	0.00	0.88	0.89	0.02	6.30	0.13	0.13	5.96	0.37	0.12	0.10	0.99	0.00
SS L	0.24	0.99	0.01	0.05	0.99	0.00	0.73	0.80	0.02	7.64	0.26	0.15	3.09	0.69	0.07
DC R	1.20	0.99	0.03	0.03	0.99	0.00	0.03	0.86	0.00	0.07	0.90	0.00	0.05	0.99	0.00
DC L	0.95	0.90	0.02	0.01	0.99	0.01	0.11	0.99	0.00	0.01	0.94	0.00	0.94	0.90	0.02
VC R	0.69	0.82	0.02	1.41	0.97	0.03	1.41	0.97	0.00	12.53	0.56	0.22	0.26	0.99	0.01
VC L	2.26	0.99	0.05	3,.47	0.55	0.07	4.05	0.86	0.08	0.94	0.99	0.02	0.00	0.98	0.00

*DC R, dorsal cingulum right hemisphere; DC L, dorsal cingulum right hemisphere; DF, degrees of freedom; FDR, false discovery rate corrected p-value; $\eta_{\text{p}}^{2}$, partial eta squared; PTR R, posterior thalamic radiation right hemisphere; PTR L, posterior thalamic radiation left hemisphere; ROI, region of interest; SS R, sagittal stratum right hemisphere; SS L, sagittal stratum left hemisphere; VC R, ventral cingulum right hemisphere; VC L, ventral cingulum left hemisphere*.

Repeated-measures ANOVA for PHQ9 showed a significant effect of time (*F*_(2,50)_ = 5.367, *p* = 0.008, $\eta_{\text{p}}^{2}$ 0.177). Paired *t*-tests showed significantly decreased depression scores from baseline to 1 week after treatment (*t*_(25)_ = 2.667, *p* = 0.013, 95%CI [4.247, 2.667], *d* = 0.41), with no significant change between 1 week and 3 months after treatment (*t*_(25)_ = −0.413, *p* = 0.683, 95%CI [−1.547, 1.030], *d* = −0.05) (Table 2).

Repeated-measures ANOVA for GAD7 showed a significant effect of time (*F*_(1,1.414,50)_ = 26.006, *p* < 0.001, $\eta_{\text{p}}^{2}$ = 0.510). Paired samples *t*-test showed significant decrease in anxiety scores from baseline to 1 week after treatment (*t*_(25)_ = 5.929, *p* < 0.001, 95%CI [2.544, 5] *d* = 0.15) with no significant change between 1 week and 3 months after treatment (*t*_(25)_ = 1.808, *p* = 0.083, 95%CI [−0.116, 1.780], *d* = 0.20) (Table 2).

Repeated-measures ANOVA in healthy controls showed no significant effects of time for depressive (*F*_(2,1.537)_ = 0.703, *p* = 0.470, $\eta_{\text{p}}^{2}$ = 0.032) or anxious symptoms (*F*_(2,42)_ = 0.151, *p* = 0.860, $\eta_{\text{p}}^{2}$ = 0.007).

### Pre-registered DTI Analyzes

TBSS analyzes did not indicate any significant differences in FA or RD between OCD patients and healthy controls at baseline or 3 months after treatment in for the ROIs. Whole-brain analyzes at uncorrected *p* < 0.001 did not reveal any significant findings.

No significant effects of group, time, or group-by-time interactions in the repeated-measures ANOVAs survived after correction for multiple comparisons in the 26 OCD patients and 22 healthy controls (Table 2).

We did not find any significant association between baseline FA in the ROIs and change in Y-BOCS 3 months after treatment using linear regression in FSL randomize or SPSS (Tables 3, 4).

**Table 3:** Linear regression results for change in mean FA with change in Y-BOCS in the OCD patients (*n* = 26).

**ROI FA**	**Beta**	**95% CI lower–upper**	***T***	**FDRp**	***r*^**2**^**
PTR R	66.70	−28.60, 162.01	1.45	0.64	0.07
PTR L	−20.75	−166.765, 125.26	−2.45	0.99	< 0.01
SS R	27.84	−158.94, 214.61	0.31	0.99	< 0.01
SS L	30.75	−126.08, 187.58	0.41	0.99	0.01
DC R	82.49	0.59, 164.49	2.08	0.38	0.13
DC L	−3.42	−92.47, 85.63	−0.080	0.94	< 0.01
VC R	−41.68	−122.04, 38.68	−1.07	0.78	0.04
VC L	7.94	−74.78, 90.67	0.20	0.94	< 0.01

*DC R, dorsal cingulum right hemisphere; DC L, dorsal cingulum right hemisphere; FDR, false discovery rate corrected p-value; PTR R, posterior thalamic radiation right hemisphere; PTR L, posterior thalamic radiation left hemisphere; R2, r squared; SS R, sagittal stratum; ROI, region of interest; SS R, sagittal stratum right hemisphere; SS L, sagittal stratum left hemisphere; T, variance; VC R, ventral cingulum right hemisphere; VC L, ventral cingulum left hemisphere; 95% CI lower–upper, 95% confidence interval*.

**Table 4:** Linear regression results for change in mean RD with change in Y-BOCS in the OCD patients (*n* = 26).

**ROI RD**	**Beta**	**95% CI lower–upper**	***T***	**FDRp**	***r*^**2**^**
PTR R	−14.84	−41.68, 12.01	−1.14	0.71	0.05
PTR L	−22.02	−96.92, 52.89	−0.61	0.73	0.01
SS R	−101.65	−248.66, 45.37	−1.43	0.66	0.07
SS L	3.99	−78.31, 86.29	0.10	0.92	< 0.01
DC R	−116.24	−214.18, −18.29	−2.45	0.18	0.17
DC L	−48.78	−166.44, 68.89	−0.86	0.80	0.03
VC R	−23.05	−80.36, 34.26	−0.83	0.66	0.02
VC L	−16.87	−81.68, 47.94	−0.54	0.68	0.01

*DC R, dorsal cingulum right hemisphere; DC L, dorsal cingulum right hemisphere; FDR, false discovery rate corrected p-value; PTR R, posterior thalamic radiation right hemisphere; PTR L, posterior thalamic radiation left hemisphere; R2, r squared; SS R, sagittal stratum; ROI, region of interest; SS R, sagittal stratum right hemisphere; SS L, sagittal stratum left hemisphere; T, variance; VC R, ventral cingulum right hemisphere; VC L, ventral cingulum left hemisphere; 95% CI lower–upper, 95% confidence interval*.

Linear regression models did not find a significant relation between illness onset before vs. after 18 years of age and FA in left (beta = 0.132, 95% CI [−0.023, 0.43], *p* = 0.748) or right sagittal stratum (beta = 0.260, 95% CI [0.008, 0.45], *p* = 0.168). Age was entered into both models as a covariate, and was not significantly related to FA in left (beta = 0.132, 95% CI [−0.002, 0.001], *p* = 0.500) and significantly related to FA in right sagittal stratum (beta = −0.564, 95% CI [−0.004, −0.001], *p* = 0.005

#### Exploratory Analyzes

**Figure 3:**
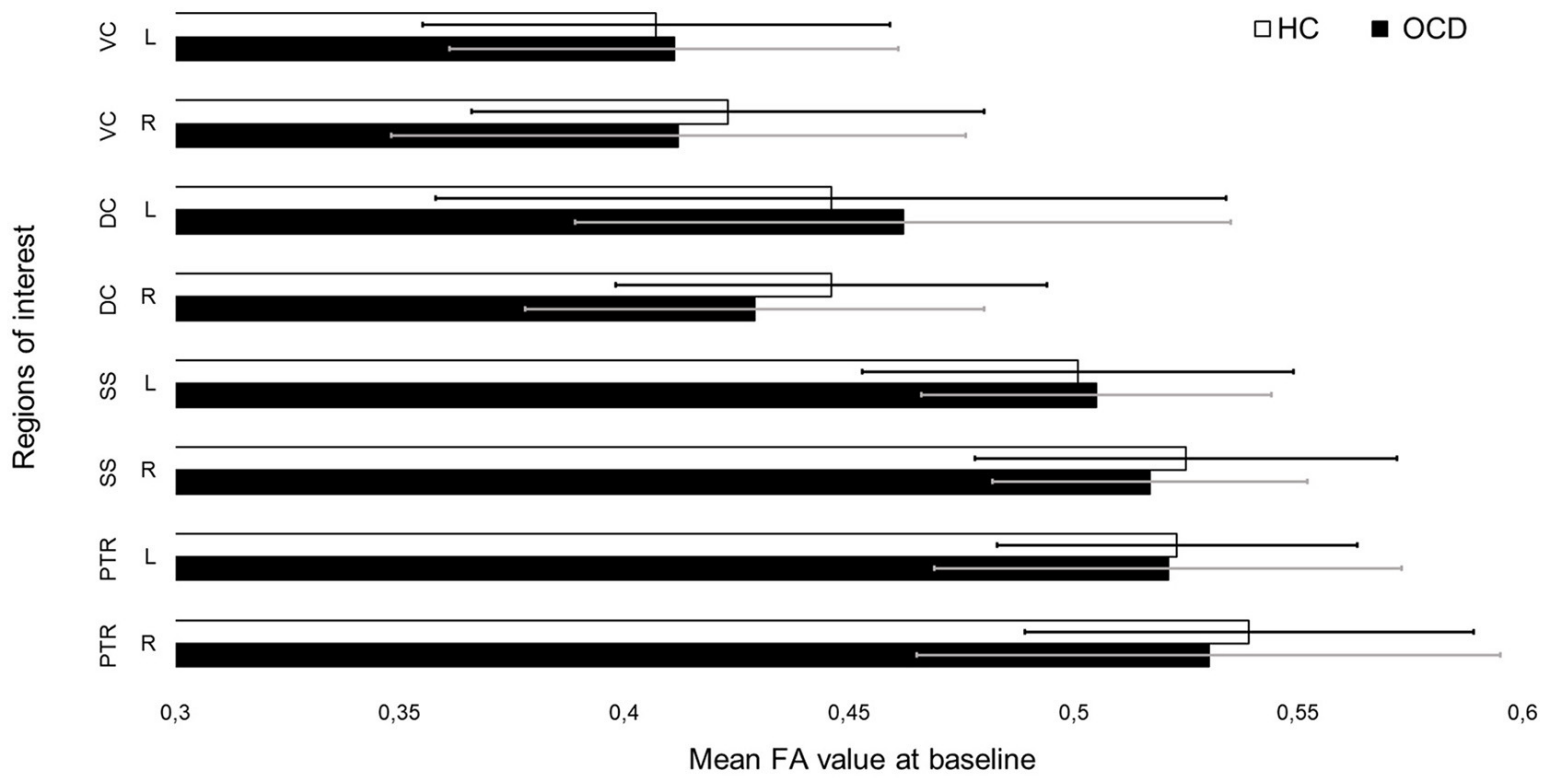
Mean fractional anisotropy (FA) values for each region of interest in patients and controls before treatment.

**Figure 4:**
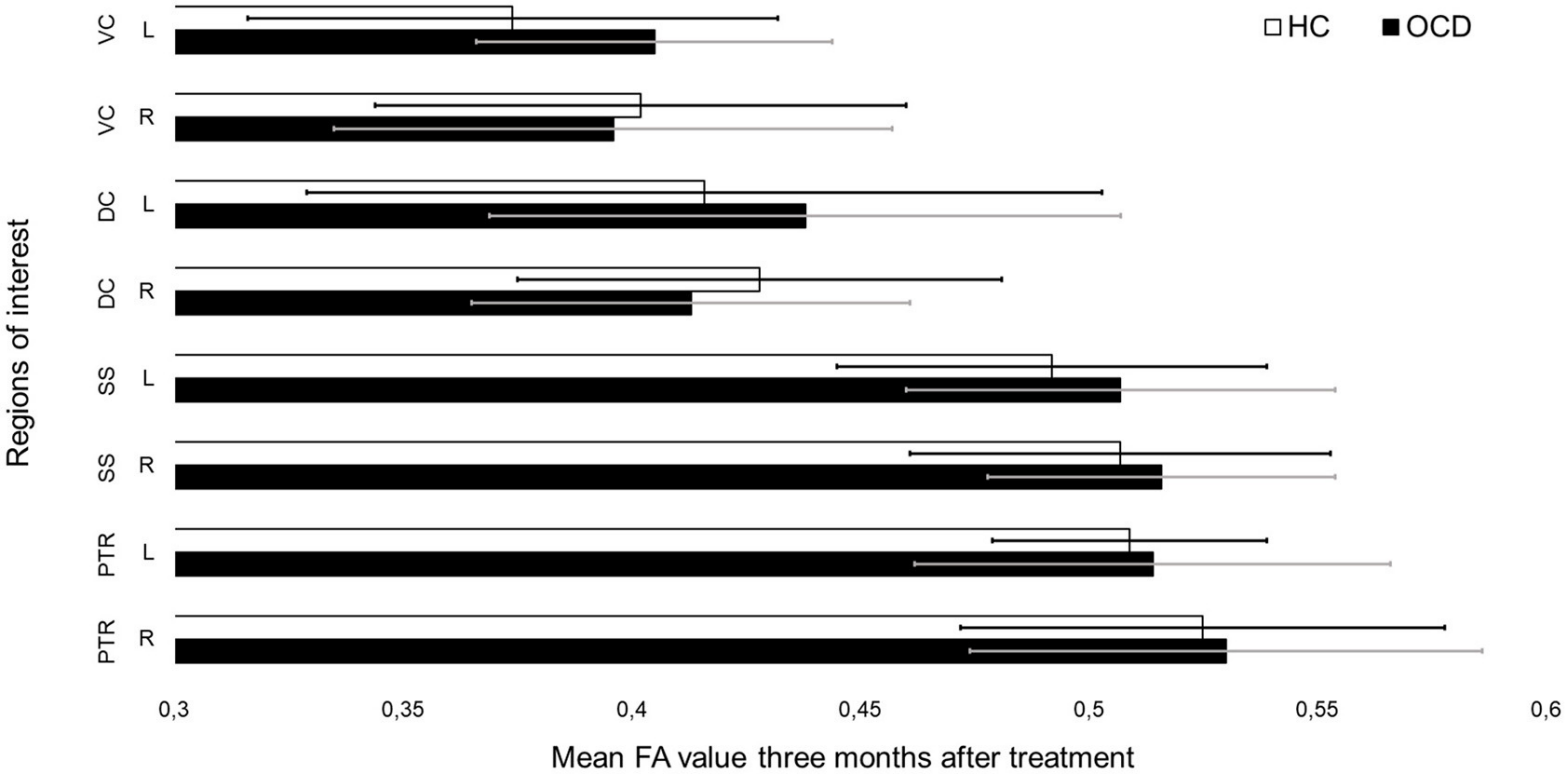
Mean fractional anisotropy (FA) values for each region of interest in patients and controls after treatment.

In SPSS, we compared the extracted means for AD, MD, and RD in the ROI in OCD patients vs. healthy controls at baseline and after months (see Figures 3 and 4 for mean FA). None of the results survived after correcting for multiple comparisons (see Supplementary Tables 1–11).

#### Sensitivity Analyzes

A series of *t*-tests were performed to investigate if comorbid anxiety or mood disorders and medication status in the OCD patients were related to FA in any of the ROI, but no tests were significant after FDR-correction (see Supplementary Tables 12–14 for results).

## Discussion

The present longitudinal study investigated white matter microstructure in OCD patients before concentrated ERP and 3 months after treatment and compared these white matter integrity measures in patients with healthy controls re-scanned at the same time point. Contrary to our hypotheses, we did not find any significant differences in mean FA or RD between OCD patients and controls in the bilateral posterior thalamic radiation, sagittal stratum, dorsal and ventral cingulum at baseline or 3 months after treatment. We did not find any significant differences in FA in the sagittal stratums between patients with illness onset before vs. after 18 years of age. The clinical results showed that 77% of the patients were in remission 3 months after treatment. Contrary to our hypotheses, the OCD sample did not present white matter alterations before treatment. The planned repeated-measures ANOVAs where nevertheless carried out to determine if stable group differences emerged with the greater statistical power afforded by two time points, or if OCD patients would show any compensatory changes. However, we found no significant evidence of either. Therefore, we conclude that white matter measures did not change after successful treatment response. Our exploratory analysis did not find influence on the results when adjusting for medication or comorbid anxiety or mood disorders. Analysis on AD, MD and RD did not reveal any group differences before or after treatment after correcting for multiple comparisons.

In contrast to previous cross-sectional studies and meta-analyzes, we did not find any group differences in the white matter tracts. Similar to the previous treatment studies, the meta-analyzes of OCD patients vs. controls differ in study inclusion and how images were processed. Piras and colleagues (5) applied meta-analyzes on both published and unpublished data in the ENIGMA OCD working group. Here, DWI were first processed and analyzed in FSL using a common pipeline across sites. They then submitted summary statistics per tract to meta-analysis. In comparison, Radua et al. (10) performed meta-analysis on published data using voxel-based analysis (VBA) and found markedly more widespread FA abnormalities. Interesting to note, a recent meta-analysis found that studies on OCD using TBSS reported fewer significant findings compared to those applying VBA on DTI (49). However, the report of less significant findings when applying TBSS contra VBM suggests that the location and magnitude of white matter abnormalities in OCD may be influenced by several factors, including the choice of image processing and analysis method.

Psychological and pharmacological treatment studies in OCD using DTI investigating changes in white matter microstructure after treatment have produced mixed results as summarized in the introduction. Thus, there seem to be few common findings (5, 8–10, 13, 25). This, together with the null findings of the present paper, suggests that changes in white matter microstructure after treatment are subtle and require large sample sizes to find significant effects. However, variation in rates of comorbidity, medication use, and symptom severity in the present study compared to previous studies may also contribute to the spurious results.

Besides variability in clinical, demographical and data processing methods between studies, the biological non-specificity of DTI limits most studies. For example, DTI cannot separate intra- and extracellular restricted diffusion, and is limited in regions with crossing, diverging and converging fibers (3, 4, 10). Drawn from our DTI results, we suggest that successful treatment may not depend on or lead to major changes in white matter detectable by the applied method. Future studies could apply multi-shell diffusion-weighted imaging in combination with advanced diffusion models, which are better able to separate crossing fibers and thus may reflect the biological processes more accurately (6).

Various usage of image processing and statistical analysis tools is a challenge in comparing previous results and the present study. The three previous treatment studies directly applied image registration to a standard space separately for each subject and time point, followed by voxel-based analysis using lenient statistical thresholds (8, 9, 25). The use of lenient threshold might signal how the changes are likely subtle. Voxel-based morphometry is found to have a higher risk of poor registration than TBSS. This is often solved by smoothing the FA images, which results in less anatomical precision (42). The use of TBSS may alleviate some of these issues, although there is some evidence that the default settings and normalization to standard space using FA images may not be optimal (43). We therefore applied image registration using full tensor information in DTI-TK, which has been shown to result in fewer misregistered voxels in white matter (43). Furthermore, image registration methods that do not account for the longitudinal nature of the data may result in poorer overlap in standard space, which may further result in spurious findings (40, 43). We therefore applied a validated longitudinal pipeline, which has been shown to result in better registration and higher test-retest reliability (41).

The present study is limited by its sample size, which although comparable to previous treatment studies, is not powered to detect the subtle differences between OCD patients and healthy controls. The study may also be underpowered to answer our secondary hypothesis that patients with an illness onset before 18 years of age would show lower FA in the sagittal stratum. Another limitation for finding predictors of treatment outcome is the high rates of remission, which reduces the explainable variation. However, this makes the study more likely to detect the hypothesized changes in the OCD patients 3 months after treatment on a group-level. Furthermore, the lack of a waiting-list group prevents us from comparing the natural course of white matter microstructure to that concentrated ERP treatment in OCD.

In conclusion, our results suggest that successful concentrated ERP may not lead to or depend on major changes in white matter microstructure detectable by DTI. However, the small sample size may have hindered the detection of subtle baseline group differences and changes over time. Taken together with previous mixed results, we suggest that larger sample sizes, rigorous analyzes, and high quality imaging processing is needed to reliably detect white matter microstructural differences between patients and controls, as well as changes after treatment.

## Data Availability Statement

The datasets for the study are not publicly available due to the restriction by Norwegian legislation for privacy protection.

## Ethics Statement

The studies involving human participants were reviewed and approved by Regional Ethics Committee for South-Eastern Norway (2015/936). The patients/participants provided their written informed consent to participate in this study.

## Author Contributions

VB: conceptualization, writing—original draft, writing—review and editing, pre-processing, formal analysis data curation, and visualization. AT: conceptualization, software, formal analysis, investigation, data curation, writing—original draft, writing—review and editing, visualization, supervision, and project administration. OO: conceptualization, writing—original draft, writing—review and editing, and supervision. CV: conceptualization, methodology, software, pre-processing, supervision, writing—original, review and editing. DA: writing—review and editing. KH: investigation, supervision, writing—original draft, writing—review and editing, supervision, and project administration. GK: conceptualization, supervision, writing—original draft, writing—review and editing, supervision, project administration, and funding acquisition. BH: conceptualization, writing—original draft, writing—review and editing, supervision, and project administration. OH: conceptualization, methodology, validation, writing—original draft, writing—review and editing, supervision, and project administration. All authors contributed to the article and approved the submitted version.

## Conflict of Interest

OH has received a speaker's honorarium from Benecke and consultation honorarium from Lundbeck. The remaining authors declare that the research was conducted in the absence of any commercial or financial relationships that could be construed as a potential conflict of interest.
