# Supplementary material for: Diffusion Tensor Imaging Before and 3 Months After Concentrated Exposure Response Prevention in Obsessive-Compulsive Disorder
Source: Front Psychiatry. 2021 May 26;12:674020. doi: 10.3389/fpsyt.2021.674020 (PMC8187597; doi:10.3389/fpsyt.2021.674020)
Supplement: Supplementary file 1 [file Data_Sheet_1.PDF]

7. Supplementary Material

Information on missing data

One week after treatment one patient did not complete the Y-BOCS interview, and four did not report GAD7 or PHQ9. Three months after treatment one patient did not attend the Y-BOCS interview, two patients were missing GAD-7, and three patients were missing PHQ-9. One healthy control was missing the GAD7 and PHQ9 at baseline and after one week, while two healthy controls were missing the GAD7 and PHQ9 after three months.

Supplemental Table 1: *Mean FA values at baseline and independent samples t-test results for OCD patients and healthy controls (HC)*

		OCD (n= 32)		HC (n = 30)							
**ROI**	**H**	**M**	**SD**	**M**	**SD**	**MD**	**F**	**t**	**FDRp**	**95% CI Lower - Upper**	***d***
PTR	R	0.53	0.07	0.54	0.05	0.02	0.02	0.65	0.83	-0.02, 0.04	0.17
PTR	L	0.52	0.05	0.52	0.04	0.01	0.00	0.16	0.87	-0.02, 0.03	0.04
SS	R	0.52	0.04	0.53	0.05	0.01	0.58	0.80	0.99	-0.01, 0.03	0.22
SS	L	0.51	0.04	0.50	0.05	0.01	0.73	-0.34	0.85	-0.03, 0.02	0.09
DC	R	0.43	0.05	0.45	0.05	0.01	0.14	1.32	0.99	-0.01, 0.04	0.34
DC	L	0.46	0.07	0.45	0.09	0.02	1.11	-0.79	0.99	-0.06, 0.03	0.21
VC	R	0.41	0.06	0.42	0.06	0.02	0.56	0.76	0.90	-0.02, 0.04	0.20
VC	L	0.41	0.05	0.41	0.05	0.01	0.01	-0.33	0.99	-0.03, 0.02	0.10

Abbreviations: d: Cohen’s d effect size; DC: dorsal cingulum; H: hemisphere; ROI: Region of interest; M: mean value; MD: mean difference; PTR: Posterior thalamic radiation; SD: Standard deviation, SS: sagittal stratum; VC: ventral cingulum

Supplemental Table 2: Mean *FA values at follow-up and independent samples t-test results for OCD patients and healthy controls (HC)*

		OCD (n=26)		HC (n=22)							
**ROI**	**H**	**M**	**SD**	**M**	**SD**	**MD**	**F**	**t**	**FDRp**	**95% CI Lower - Upper**	***d***
PTR	R	0.53	0.06	0.53	0.05	-0.01	0.13	-0.35	0.73	-0.04, 0.03	0.09
PTR	L	0.51	0.05	0.51	0.03	-0.01	1.07	-0.43	0.89	-0.03, 0.02	0.02
SS	R	0.52	0.04	0.51	0.05	-0.01	0.56	-0.76	0.72	-0.03, 0.02	0.21
SS	L	0.51	0.05	0.49	0.05	-0.02	0.10	-1.08	0.99	-0.04, 0.01	0.32
DC	R	0.41	0.05	0.43	0.05	0.02	0.38	1.03	0.83	-0.01, 0.04	0.30
DC	L	0.44	0.07	0.42	0.09	-0.02	0.72	-0.99	0.66	-0.07, 0.02	0.28
VC	R	0.40	0.06	0.40	0.06	0.01	0.28	0.37	0.81	-0.03, 0.04	0.10
VC	L	0.41	0.04	0.37	0.06	-0.03	4.26	-2.16	0.32	-0.06, -0.02	0.63

Abbreviations: d: Cohen's d effect size; DC: dorsal cingulum; H: hemisphere; ROI: Region of interest; M: mean value; MD: Mean difference; PTR: Posterior thalamic radiation; SD: Standard deviation, SS: sagittal stratum; VC: ventral cingulum.

Supplemental Table 3: *Mean AD values at baseline and independent samples t-test results for OCD patients and healthy controls (HC)*

		OCD (n= 32)		HC (n = 30)							
**ROI**	**H**	**M**	**SD**	**M**	**SD**	**MD**	**F**	**t**	**FDRp**	**95% CI Lower - Upper**	***d***
PTR	R	1.34	0.19	1.33	0.11	-0.01	0.66	-0.25	0.99	-0.09, 0.07	0.06
PTR	L	1.37	0.16	1.34	0.19	0.04	2.65	1.22	0.99	-0.03, 0.11	0.17
SS	R	1.33	0.09	1.37	0.16	0.00	0.02	0.23	0.94	-0.03, 0.04	0.31
SS	L	1.31	0.06	1.33	0.09	0.02	1.39	0.77	0.88	-0.02, 0.06	0.26
DC	R	1.31	0.06	1.31	0.06	0.01	0.03	0.73	0.75	-0.02, 0.04	0.33
DC	L	1.32	0.09	1.31	0.06	-0.02	0.00	-1.14	0.99	-0.06, 0.02	0.13
VC	R	1.31	0.07	1.32	0.09	0.02	1.13	1.05	0.80	-0.02, 0.07	0.25
VC	L	1.15	0.06	1.31	0.07	0.00	0.45	0.05	0.96	-0.03, 0.03	2.45

Abbreviations: d: Cohen's d effect size; DC: dorsal cingulum; H: hemisphere; ROI: Region of interest; M: mean value; MD: mean difference; PTR: Posterior thalamic radiation; SD: Standard deviation, SS: sagittal stratum; VC: ventral cingulum

Supplemental Table 4: *Repeated measures ANOVA results for mean AD in OCD patients (n=26) and healthy controls (n=22)*

	Time			Group			Time by group			Age			Gender		
**ROI**	**F**	**FDRp**	η^2_p	**F**	**FDRp**	η^2_p	**F**	**FDRp**	η^2_p	**F**	**FDRp**	η^2_p	**F**	**FDRp**	η^2_p
PTR R	1.371	0.84	0.03	0.15	0.70	0.00	2.88	0.64	0.06	1.21	0.73	0.03	3.04	0.64	0.07
PTR L	0.67	0.99	0.02	1.74	0.99	0.04	0.74	0.99	0.02	2.44	0.89	0.05	0.00	0.99	0.00
SS R	0.16	0.99	0.00	0.16	0.69	0.00	1.36	0.99	0.03	2.72	0.72	0.06	2.74	0.99	0.06
SS L	0.10	0.89	0.00	0.91	0.99	0.02	0.79	0.99	0.02	6.27	0.99	0.13	2.61	0.99	0.06
DC R	0.03	0.48	0.00	0.05	0.83	0.00	0.00	0.87	0.00	0.32	0.83	0.01	0.00	0.38	0.00
DC L	276.60	0.64	0.86	0.91	0.35	0.02	0.24	0.74	0.01	0.01	0.66	0.01	0.45	0.94	0.01
VC R	0.02	0.78	0.00	1.49	0.83	0.00	0.04	0.78	0.00	0.86	0.81	0.02	5.12	0.78	0.10
VC L	0.98	0.94	0.00	0.09	0.77	0.00	3.83	0.90	0.08	4.37	0.32	0.09	0.00	0.94	0.00

Abbreviations: DC R: dorsal cingulum right hemisphere; DC L: dorsal cingulum right hemisphere; DF: degrees of freedom; FDR: False Discovery Rate corrected p-value; η^2_p: partial eta squared; PTR R: Posterior thalamic radiation right hemisphere; PTR L: posterior thalamic radiation left hemisphere; ROI: region of interest; SS R: sagittal stratum right hemisphere; SS L: sagittal stratum left hemisphere; VC R: ventral cingulum right hemisphere; VC L: ventral cingulum left hemisphere.

Supplemental Table 5: Mean *AD values at follow-up and independent samples t-test results for OCD patients and healthy controls (HC)*

		OCD (n=26)		HC (n=22)							
**ROI**	**H**	**M**	**SD**	**M**	**SD**	**MD**	**F**	**t**	**FDRp**	**95% CI Lower - Upper**	***d***
PTR	R	1.33	0.14	1.33	0.11	0.00	0.72	-0.01	0.99	-0.08, 0.08	0
PTR	L	1.34	0.15	1.33	0.14	0.04	1.33	0.97	0.91	-0.04, 0.11	0.07
SS	R	1.31	0.11	1.34	0.15	-0.02	0.15	-1.02	0.99	-0.06, 0.02	0.23
SS	L	1.28	0.06	1.31	0.11	0.01	2.78	0.25	0.99	-0.04, 0.06	0.34
DC	R	1.30	0.07	1.28	0.06	0.00	3.07	0.12	0.99	-0.03, 0.03	0.31
DC	L	1.31	0.11	1.30	0.07	-0.02	0.00	-0.79	0.69	-0.06, 0.02	0.11
VC	R	1.31	0.07	1.31	0.11	0.03	0.11	0.92	0.72	-0.03, 0.09	0
VC	L	1.12	0.04	1.31	0.07	-0.02	4.22	-1.04	0.99	-0.06, 0.02	3.33

Abbreviations: d: Cohen’s d effect size; DC: dorsal cingulum; H: hemisphere; ROI: Region of interest; M: mean value; MD: Mean difference; PTR: Posterior thalamic radiation; SD: Standard deviation, SS: sagittal stratum; VC: ventral cingulum.

Supplemental Table 6: *Mean MD values at baseline and independent samples t-test results for OCD patients and healthy controls (HC)*

		OCD (n= 32)		HC (n = 30)							
**ROI**	**H**	**M**	**SD**	**M**	**SD**	**MD**	**F**	**t**	**FDRp**	**95% CI Lower - Upper**	***d***
PTR	R	0.82	0.20	0.80	0.09	-0.02	0.53	-0.52	0.99	-0.10, 0.06	0.13
PTR	L	0.84	0.13	0.82	0.20	0.03	3.41	1.13	0.99	-0.02, 0.08	0.12
SS	R	0.81	0.06	0.84	0.13	0.00	0.00	-0.34	0.97	-0.02, 0.02	0.29
SS	L	0.80	0.04	0.81	0.06	0.01	3.24	1.02	0.99	-0.01, 0.04	0.20
DC	R	0.80	0.04	0.80	0.04	-0.01	0.08	-0.92	0.96	-0.02, 0.01	0.25
DC	L	0.83	0.06	0.80	0.04	0.00	0.27	-0.28	0.89	-0.02, 0.01	0.59
VC	R	0.82	0.03	0.83	0.06	0.01	0.14	0.37	0.99	-0.04, 0.05	0.85
VC	L	0.75	0.02	0.82	0.03	0.00	0.07	0.25	0.80	-0.02, 0.02	2.75

Abbreviations: d: Cohen’s d effect size; DC: dorsal cingulum; H: hemisphere; ROI: Region of interest; M: mean value; MD: mean difference; PTR: Posterior thalamic radiation; SD: Standard deviation, SS: sagittal stratum; VC: ventral cingulum

Supplemental Table 7: *Repeated measures ANOVA results for mean MD in OCD patients (n=26) and healthy controls (n=22)*

	Time			Group			Group by time			Age			Gender		
**ROI**	**F**	**FDRp**	η^2_p	**F**	**FDRp**	η^2_p	**F**	**FDRp**	η^2_p	**F**	**FDRp**	η^2_p	**F**	**FDRp**	η^2_p
PTR R	0.69	0.99	0.02	0.25	0.99	0.01	2.14	0.64	0.05	5.02	0.22	0.57	1.64	0.97	0.04
PTR L	0.24	0.99	0.01	2.15	0.89	0.05	1.34	0.99	0.03	0.63	0.77	0.01	0.27	0.77	0.01
SS R	0.63	0.87	0.01	0.08	0.97	0.00	0.00	0.86	0.00	0.32	0.99	0.01	1.48	0.99	0.03
SS L	0.23	0.99	0.01	1.07	0.99	0.31	0.02	0.73	0.00	0.09	0.69	0.00	0.06	0.69	0.00
DC R	0.02	0.96	0.00	1.17	0.96	0.03	0.00	0.89	0.00	0.07	0.79	0.00	0.19	0.99	0.00
DC L	0.11	0.89	0.00	0.09	0.89	0.00	1.79	0.99	0.04	0.11	0.50	0.00	1.07	0.90	0.02
VC R	0.81	0.99	0.02	0.30	0.99	0.01	0.11	0.90	0.00	1.27	0.99	0.03	0.30	0.99	0.09
VC L	0.76	0.80	0.02	0.84	0.80	0.02	0.03	0.99	0.00	1.28	0.98	0.03	0.00	0.98	0.00

Abbreviations: DC R: dorsal cingulum right hemisphere; DC L: dorsal cingulum right hemisphere; DF: degrees of freedom; FDR: False Discovery Rate corrected p-value; η^2_p: partial eta squared; PTR R: Posterior thalamic radiation right hemisphere; PTR L: posterior thalamic radiation left hemisphere; ROI: region of interest; SS R: sagittal stratum right hemisphere; SS L: sagittal stratum left hemisphere; VC R: ventral cingulum right hemisphere; VC L: ventral cingulum left hemisphere.

Supplemental Table 8: Mean *MD values at follow-up and independent samples t-test results for OCD patients and healthy controls (HC)*

		OCD (n=26)		HC (n=22)							
**ROI**	**H**	**M**	**SD**	**M**	**SD**	**MD**	**F**	**t**	**FDRp**	**95% CI Lower - Upper**	***d***
PTR	R	0.81	0.13	0.81	0.10	0.00	0.22	0.04	0.64	-0.07. 0.07	0
PTR	L	0.84	0.12	0.81	0.13	0.03	1.72	1.10	0.40	-0.03. 0.09	0.24
SS	R	0.80	0.08	0.84	0.12	0.00	0.32	-0.30	0.76	-0.03. 0.02	0.39
SS	L	0.80	0.04	0.80	0.08	0.02	2.06	0.85	0.43	-0.02. 0.06	0
DC	R	0.80	0.05	0.80	0.04	-0.01	0.28	-0.92	0.69	-0.02. 0.01	0
DC	L	0.83	0.09	0.80	0.05	0.01	4.60	0.77	0.32	-0.01. 0.03	0.41
VC	R	0.81	0.04	0.83	0.09	0.02	0.34	0.55	0.90	-0.05. 0.08	0.29
VC	L	0.75	0.03	0.81	0.04	0.01	3.63	0.76	0.24	-0.01. 0.03	1.70

Abbreviations: d: Cohen’s d effect size; DC: dorsal cingulum; H: hemisphere; ROI: Region of interest; M: mean value; MD: Mean difference; PTR: Posterior thalamic radiation; SD: Standard deviation. SS: sagittal stratum; VC: ventral cingulum.

Supplemental Table 9: *Mean RD values at baseline and independent samples t-test results for OCD patients and healthy controls (HC)*

		OCD (n= 32)		HC (n = 30)							
**ROI**	**H**	**M**	**SD**	**M**	**SD**	**MD**	**F**	**t**	**FDRp**	**95% CI Lower - Upper**	***d***
PTR	R	0.55	0.21	0.53	0.09	-0.03	0.64	-0.62	0.99	-0.11. 0.06	1.70
PTR	L	0.57	0.13	0.55	0.21	0.02	2.66	0.96	0.99	-0.03. 0.08	0.11
SS	R	0.55	0.07	0.57	0.13	-0.01	0.08	-0.56	0.77	-0.03. 0.02	0.19
SS	L	0.54	0.06	0.55	0.07	0.01	0.72	0.81	0.99	-0.02. 0.04	0.15
DC	R	0.55	0.04	0.54	0.06	-0.01	0.10	-1.43	0.99	-0.03. 0.01	0.20
DC	L	0.58	0.07	0.55	0.04	0.01	1.87	0.58	0.90	-0.02. 0.04	0.53
VC	R	0.57	0.04	0.58	0.07	0.00	0.00	0.03	0.98	-0.05. 0.05	0.18
VC	L	0.55	0.03	0.57	0.04	0.00	0.10	0.24	0.93	-0.02. 0.03	0.57

Abbreviations: d: Cohen's d effect size; DC: dorsal cingulum; H: hemisphere; ROI: Region of interest; M: mean value; MD: mean difference; PTR: Posterior thalamic radiation; SD: Standard deviation. SS: sagittal stratum; VC: ventral cingulum

Supplemental Table 10: *Repeated measures ANOVA results for mean RD in OCD patients (n=26) and healthy controls (n=22)*

	Time			Group			Group by time			Age			Gender		
**ROI**	**F**	**FDRp**	η^2_p	**F**	**FDRp**	η^2_p	**F**	**FDRp**	η^2_p	**F**	**FDRp**	η^2_p	**F**	**FDRp**	η^2_p
PTR R	0.41	0.64	0.01	0.27	0.69	0.01	1.66	0.99	0.04	7.29	0.64	0.14	0.94	0.95	0.02
PTR L	0.08	0.99	0.02	2.15	0.50	0.05	1.57	0.22	0.03	0.04	0.50	0.00	0.69	0.99	0.02
SS R	0.78	0.76	0.02	0.02	0.90	0.00	1.31	0.87	0.03	2.70	0.76	0.06	0.34	0.94	0.01
SS L	0.23	0.99	0.01	0.72	0.99	0.02	0.13	0.72	0.00	1.11	0.43	0.03	0.72	0.62	0.02
DC R	0.06	0.69	0.00	1.43	0.99	0.03	0.01	0.92	0.00	0.02	0.89	0.00	0.14	0.51	0.00
DC L	0.11	0.89	0.00	0.09	0.87	0.00	1.79	0.39	0.04	0.11	0.99	0.00	1.07	0.87	0.02
VC R	1.25	0.90	0.03	0.04	0.89	0.00	0.12	0.73	0.00	3.80	0.90	0.08	3.52	0.99	0.07
VC L	2.21	0.94	0.05	1.62	0.60	0.04	1.42	0.89	0.03	0.00	0.24	0.00	0.00	0.72	0.00

Abbreviations: DC R: dorsal cingulum right hemisphere; DC L: dorsal cingulum right hemisphere; DF: degrees of freedom; FDR: False Discovery Rate corrected p-value; η^2_p: partial eta squared; PTR R: Posterior thalamic radiation right hemisphere; PTR L: posterior thalamic radiation left hemisphere; ROI: region of interest; SS R: sagittal stratum right hemisphere; SS L: sagittal stratum left hemisphere; VC R: ventral cingulum right hemisphere; VC L: ventral cingulum left hemisphere.

Supplemental Table 11: Mean *RD values at follow-up and independent samples t-test results for OCD patients and healthy (HC)*

		OCD (n=26)		HC (n=22)							
**ROI**	**H**	**M**	**SD**	**M**	**SD**	**MD**	**F**	**t**	**FDRp**	**95% CI Lower - Upper**	***d***
PTR	R	0.55	0.14	0.55	0.10	0.00	0.07	0.07	0.95	-0.07. 0.07	0
PTR	L	0.58	0.10	0.55	0.14	0.03	0.39	1.08	0.99	-0.03. 0.08	0.25
SS	R	0.55	0.08	0.58	0.10	0.00	0.00	0.23	0.94	-0.03. 0.04	0.33
SS	L	0.56	0.06	0.55	0.08	0.02	0.36	1.02	0.62	-0.02. 0.06	0.14
DC	R	0.55	0.05	0.56	0.06	-0.01	0.69	-1.00	0.51	-0.04. 0.01	0.18
DC	L	0.59	0.09	0.55	0.05	0.02	3.64	1.06	0.77	-0.02. 0.06	0.55
VC	R	0.57	0.05	0.59	0.09	0.01	0.20	0.34	0.99	-0.06. 0.08	0.27
VC	L	0.60	0.03	0.57	0.05	0.02	3.69	1.76	0.72	0.00. 0.05	0.73

Abbreviations: d: Cohen's d effect size; DC: dorsal cingulum; H: hemisphere; ROI: Region of interest; M: mean value; MD: Mean difference; PTR: Posterior thalamic radiation; SD: Standard deviation. SS: sagittal stratum; VC: ventral cingulum.

Supplemental Table 12: Results from independent samples t-test on medication influence on baseline FA in the OCD patients

		Mood (n=11)		No mood (n=21)							
**ROI**	**H**	**M**	**SD**	**M**	**SD**	**MD**	**F**	**t**	**FDRp**	**95% CI Lower - Upper**	***d***
PTR	R	0.52	0.03	0.54	0.08	0.02	0.90	0.75	0.99	-0.03. 0.07	0.33
PTR	L	0.54	0.03	0.52	0.03	0.04	2.45	2.19	0.32	0.00. 0.08	0.67
SS	R	0.50	0.07	0.54	0.03	0.00	0.28	-0.24	0.81	-0.03. 0.02	0.74
SS	L	0.52	0.03	0.50	0.07	0.01	0.16	0.73	0.99	-0.02. 0.04	0.37
DC	R	0.52	0.04	0.52	0.03	-0.01	2.04	-0.46	0.87	-0.05. 0.03	0.57
DC	L	0.51	0.04	0.52	0.04	-0.01	0.00	-0.49	0.99	-0.07. 0.04	0.25
VC	R	0.50	0.04	0.51	0.04	0.01	2.47	0.56	0.99	-0.04. 0.06	0.25
VC	L	0.43	0.05	0.50	0.04	0.01	0.18	0.32	0.87	-0.03. 0.04	1.55

Abbreviations: d: Cohen's d effect size; DC: dorsal cingulum; H: hemisphere; ROI: Region of interest; M: mean value; MD: mean difference; PTR: Posterior thalamic radiation; SD: Standard deviation. SS: sagittal stratum; VC: ventral cingulum.

Supplemental Table 13: Results from independent samples t-test on comorbid anxiety influence on baseline FA in the OCD patients

		Anxiety (n=15)		No anxiety (n=17)							
**ROI**	**H**	**M**	**SD**	**M**	**SD**	**MD**	**F**	**t**	**FDRp**	**95% CI Lower - Upper**	***d***
PTR	R	0.51	0.09	0.54	0.04	0.03	2.99	1.26	0.99	-0.02. 0.08	0.43
PTR	L	0.53	0.03	0.51	0.09	0.01	1.94	0.54	0.94	-0.03. 0.05	0.30
SS	R	0.52	0.07	0.53	0.03	0.00	2.32	0.36	0.96	-0.02. 0.03	0.19
SS	L	0.52	0.03	0.52	0.07	0.01	0.66	0.75	0.92	-0.02. 0.04	0
DC	R	0.51	0.04	0.52	0.03	-0.02	0.05	-1.15	0.99	-0.06. 0.02	0.28
DC	L	0.51	0.04	0.51	0.04	-0.01	0.82	-0.28	0.89	-0.06. 0.05	0
VC	R	0.50	0.04	0.51	0.04	-0.02	0.47	-1.05	0.80	-0.07. 0.02	0.25
VC	L	0.42	0.05	0.50	0.04	0.00	0.25	-0.13	0.90	-0.04. 0.03	1.77

Abbreviations: d: Cohen’s d effect size; DC: dorsal cingulum; H: hemisphere; ROI: Region of interest; M: mean value; MD: Mean difference; PTR: Posterior thalamic radiation; SD: Standard deviation. SS: sagittal stratum; VC: ventral cingulum.

Supplemental Table 14: Results from independent samples t-test on medication influence on baseline FA in the OCD patients

		**Medicated (n=8)**		**Not medicated (n=24)**							
**ROI**	**H**	**M**	**SD**	**M**	**SD**	**MD**	**F**	**t**	**FDRp**	**95% CI Lower - Upper**	***d***
PTR	R	0.55	0.03	0.52	0.07	-0.03	1.36	-1.01	0.85	-0.08. 0.03	0.56
PTR	L	0.52	0.06	0.55	0.03	-0.02	0.06	-0.86	0.80	-0.06. 0.03	0
SS	R	0.54	0.04	0.52	0.06	0.00	1.32	0.31	0.87	-0.03. 0.03	0.39
SS	L	0.52	0.03	0.54	0.04	0.00	1.98	-0.08	0.94	-0.03. 0.03	0.57
DC	R	0.51	0.05	0.52	0.03	-0.03	0.11	-1.23	0.92	-0.07. 0.02	0.24
DC	L	0.50	0.04	0.51	0.05	-0.02	1.34	-0.57	0.91	-0.08. 0.04	0.22
VC	R	0.51	0.04	0.50	0.04	0.01	2.16	0.37	0.96	-0.04. 0.06	0.25
VC	L	0.42	0.05	0.51	0.04	-0.03	0.93	-1.30	0.29	-0.07. 0.01	1.99

Abbreviations: d: Cohen's d effect size; DC: dorsal cingulum; H: hemisphere; ROI: Region of interest; M: mean value; MD: Mean difference; PTR: Posterior thalamic radiation; SD: Standard deviation. SS: sagittal stratum; VC: ventral cingulum.
